# Novel Pattern of Nuclear Staining With ROS1 Immunohistochemistry: A Case Report

**DOI:** 10.1155/crpu/2900967

**Published:** 2026-06-02

**Authors:** Diane M. Wilcock, Deepika Sirohi, Kristina Moore, Allie H. Grossmann, Georgios Deftereos

**Affiliations:** ^1^ The Institute for Experimental Pathology, ARUP Laboratories, Salt Lake City, Utah, USA, aruplab.com; ^2^ Department of Pathology, University of California, San Francisco, San Francisco, California, USA, ucsf.edu; ^3^ Clinical Cancer Genomics Laboratory, University of California, San Francisco, San Francisco, California, USA, ucsf.edu; ^4^ Department of Pathology, University of Utah and ARUP Laboratories, Salt Lake City, Utah, USA; ^5^ Providence Portland Medical Center, Portland, Oregon, USA

**Keywords:** FISH, HCC-78 cell line, IHC, lung adenocarcinoma, nonsmall cell lung cancer, nuclear staining 5^′^ deletion, ROS1

## Abstract

*ROS1* rearrangements are driver events in a subset of nonsmall cell lung carcinomas and may qualify patients for *ROS1*‐directed treatment. ROS1 immunohistochemistry (IHC) is often used for screening of lung carcinomas for *ROS1* gene rearrangement and has a cytoplasmic and/or membranous staining pattern. Here, we report an unusual case of a lung adenocarcinoma displaying a strong nuclear staining pattern on ROS1 IHC. *ROS1* fluorescence in situ hybridization (FISH) reflex testing was ordered. ROS1 FISH testing showed an isolated 3 ^′^ signal pattern, indicating a potential rearrangement or deletion in the ROS1 gene. Next‐generation sequencing (NGS) analysis revealed a *NMP1::ROS1* rearrangement. The tumor was from a 74‐year‐old male with a previous history of lung squamous cell carcinoma. This new occurrence was determined to be a lung adenocarcinoma and displayed papillary features, whereas concurrent lymphadenopathy was also present. Immunostaining for ALK was negative, and there was no expression of PD‐L1 22C3 IHC. This case report highlights an unexpected ROS1 IHC staining associated with a rare *ROS1* gene rearrangement.

## 1. Introduction

*ROS1* gene rearrangements occur in approximately 1%–2% of nonsmall cell lung cancers (NSCLCs), and largely in lung adenocarcinomas [1]. Tumors with rearrangement of the *ROS1* gene are targetable by tyrosine kinase inhibitors like crizotinib [2]. ROS1 immunohistochemistry (IHC) is routinely used to screen lung cancers for *ROS1* rearrangements with FISH being used as the confirmatory method [3].

Although ROS1 IHC typically displays a cytoplasmic staining pattern, there have been some reports of staining variations. *ROS1* has been reported to have a number of different fusion partners, with *CD74*, EZR, SDC4, FIG, SLC34A2, and TPM3 being the most common [4–6]. Subcellular localization and downstream signaling may differ depending on the fusion partner of ROS1. For instance, *EZR::ROS1* fusions can be associated with plasma membranous accentuation on ROS1 IHC slides [4, 5] *CD74::ROS1* fusions can have more of a focal globular immunoreactivity [4]. Strong perinuclear aggregates as well as membrane and vesicular localization of ROS1 have also been reported [6]. Outside of nonsmall cell lung carcinomas, different patterns of ROS1 IHC staining have also been described in the context of inflammatory myofibroblastic tumors [7].

These variant staining patterns highlight that ROS1 staining can be complex. The strong nuclear ROS1 staining in the case presented is a variant pattern that may be associated with *ROS1* gene rearrangement and therefore needs to be further investigated.

## 2. Case Presentation

The patient was a 74‐year‐old male with a previous history of lung squamous cell carcinoma who presented with a new right lower lobe lung mass and mediastinal lymphadenopathy. A transbronchial biopsy was performed and showed an adenocarcinoma with papillary features, which was positive for TTF‐1 and napsin‐A, consistent with a lung primary (immunostains performed at referring laboratory). IHC and FISH testing was performed at ARUP Laboratories (Salt Lake City, Utah). IHC staining for ALK was performed on an automated Ventana Benchmark stainer (Roche, Rotkreuz, Switzerland) using rabbit primary monoclonal ALK D5F3 antibody (Cell Signaling Technology, Danvers, Massachusetts, United States) at a 1:100 dilution with incubation for 1 h at 37°C. IHC for ROS1 was performed on an automated Leica Bond III stainer (Leica Biosystems, Deer Park, Illinois, United States) using rabbit primary monoclonal ROS1 D4D6 antibody (Cell Signaling Technology, Danvers, Massachusetts, United States) at a 1:50 dilution, with incubation for 1 h at 37°C. Appropriate positive and negative controls were run alongside the patient slides.

**Figure 1:**
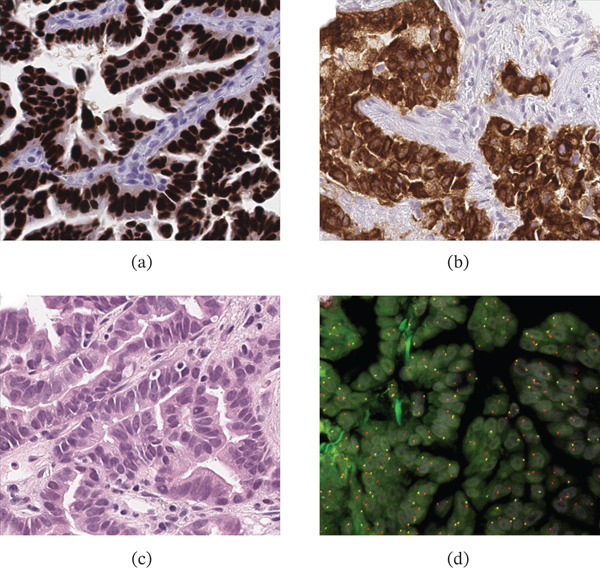
ROS1 IHC and FISH of this case, highlighting the difference in IHC patterns between this case and usual ROS1 IHC positive staining pattern. (a) ROS1 IHC displaying nuclear staining in this patient′s case, 40×. (b) Typical ROS1 IHC staining, separate clinical case, 40×. (c) H&E slide on ROS1 nuclear IHC staining case, 40×. (d) ROS1 FISH displaying normal fusion signals (yellow) and isolated 3 ^′^ signal (red) in this patient′s sample, 100×.

The tumor cells displayed ROS1 IHC staining, which was primarily located in the nucleus, as shown in Figure 1a. This is different from the expected membranous and cytoplasmic staining (Figure 1b, with Figure 1c providing morphological reference on H&E staining), and this unexpected staining pattern was confirmed upon repeat testing. Confirmatory *ROS1* FISH testing showed an isolated 3 ^′^ signal present in 85% of the tumor cells, which for this assay has been validated to be consistent with *ROS1* gene rearrangement (Figure 1d). ALK IHC was negative, and PD‐L1 22C3 IHC showed a tumor proportion score (TPS) of < 1%. ROS1 IHC was repeated per pathologist request to confirm the staining pattern. The ROS1 IHC and FISH positive control was the HCC‐78 cell line with a known SLC3A4‐ROS1 rearrangement (ATCC–American Type Culture Collection, Manassas, Virginia) [8], and it displayed the expected cytoplasmic staining pattern [9], as did the other clinical samples on both the original and repeat run. The ROS1 IHC negative control was skin tissue and appropriately showed no staining. The ROS1 FISH negative control was renal tissue and appropriately showed no rearrangement. IHC for PD‐L1 was performed on a Dako Autostainer Link 48 using the PD‐L1 IHC 22C3 PharmDx kit (both Agilent Technologies, Santa Clara, California, United States) as per the manufacturer′s instructions.

Confirmatory *ROS1* FISH was performed on a slide that was pretreated on a DAKO PT Link and then underwent a pepsin digestion step (Agilent Technologies) on a VIP2000. The slide was then probed with an *ROS1* Break‐apart probe (Agilent Technologies, Cat# G111401). The slide was then denatured and hybridized on a ThermoBrite FISH slide processing system (Leica Biosystems, Deer Park, Illinois, United States). Following hybridization, slides were washed with stringency buffer (Agilent Technologies) and dehydrated through a series of ethanol solutions. They were counterstained with Vectashield Antifade Mounting Medium with DAPI (Vector Laboratories, Newark, California, United States) and coverslipped. One‐hundred tumor nuclei were enumerated. The tumor cells exhibited an unusual pattern, with most showing one fusion signal and one isolated 3 ^′^ signal, suggestive of *ROS1* rearrangement.

**Figure 2:**
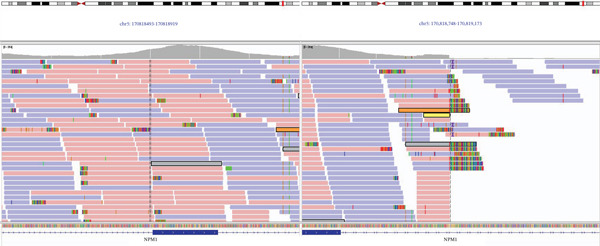
Next‐generation sequencing results, highlighting the supporting reads that confirm the *NPM1::ROS1* fusion.

As such, further investigation was deemed necessary to elucidate any possible structural rearrangement involving *ROS1* in this patient′s case. Comprehensive genomic profiling, including testing for *ROS1* rearrangements was performed at the Clinical Cancer Genomics Laboratory, University of California San Francisco (San Francisco, California, United States). Genomic DNA was extracted from macrodissected, formalin‐fixed, paraffin‐embedded (FFPE) slides form this specimen, using the QIAamp DNA FFPE Tissue Kit (Qiagen, Hilden, Germany) according to the manufacturer′s protocol. Capture‐based next‐generation DNA sequencing was performed using the UCSF500 assay. This assay targets all coding exons of 529 cancer‐related genes, in addition to select introns and upstream regulatory regions of 73 genes (including *ROS1*) to enable detection of structural variants including gene fusions, and DNA segments at regular intervals along each chromosome to enable genome‐wide copy number and zygosity analysis, with a total sequencing footprint of 2.8 Mb, and is run on the NovaSeq 6000 Sequencing System (Illumina, San Diego, California, United States). Comprehensive genomic profiling revealed an in‐frame rearrangement involving Intron 5 of NMP1 and Intron 34 of ROS1, supported by 13 reads, identified in both read directions (Figure 2).

## 3. Discussion

To the best of our knowledge, this is the first report of nuclear ROS1 IHC staining in any tumor type. The rarity of this finding is emphasized by the fact that it was detected during routine testing in a large reference laboratory setting (ARUP Laboratories, Salt Lake City, Utah). ROS1 IHC is a high‐volume test, driven by the clinical actionability of ROS1 fusions in highly prevalent NSCLC. The clinical significance of nuclear ROS1 staining in NSCLC lies in awareness of such staining pattern that should be confirmed by another method such as FISH and/or sequencing.

The isolated 3 ^′^ FISH pattern our sample displayed is interesting. The kinase domain for ROS1 is located in the 3 ^′^ end of the gene [10]. This means that retention of the 3 ^′^ probe may lead to dysregulated kinase activity. The isolated 3 ^′^ signal pattern is a common finding in break‐apart FISH with this probe set. For example, one study that evaluated in situ and extraction‐based methods for *ROS1* rearrangements found that 21 out of 32 *ROS1* FISH positive cases demonstrated an isolated 3 ^′^ signal pattern and 10 of the samples could be confirmed by at least two other molecular methods [11]. The ROS1 D4D6 antibody reacts with the carboxy‐terminal domains of the ROS1 protein. As such, the IHC was able to detect the fusion protein and its aberrant nuclear localization, which was our first clue to this rare fusion.

It has been shown that ROS1 protein localization to distinct cellular compartments may be influenced by its fusion partner. For example, *SLC34A3::ROS1* localizes to the paranuclear compartment [12]. In contrast, *FIG::ROS1* fusions expression is seen in the golgi apparatus, and these tumors have been shown to be less invasive with lesser metastatic potential than tumors that have localization of ROS1 to the cell membrane via a CD74 fusion [12, 13]. This may be due to phosphorylation of plasma membrane protein E‐Syt1 mediated by the CD74‐ROS1 fusion protein but not by the FIG‐ROS1 fusion protein [13] The functional impact of a ROS1 fusion protein localizing to the nucleus is unknown. However, it could potentially lead to activation of a different set of downstream targets as seen with the *FIG-ROS1* fusion protein. Additional cases of ROS1 nuclear staining will be needed to define this unique subset more fully.

Although not common, *NPM1::ROS1* rearrangements have been rarely reported in literature [14, 15]. *NPM1* encodes for the nucleophosmin, an abundant protein that shuttles between the nucleus and cytoplasm but localizes mainly in the nucleolus [16]. This may explain the aberrant nuclear localization of the ROS1 protein in this case. Where available, the data suggest that the breakpoints seen in *ROS1* commonly involve Exons 35–43 [14], which is inclusive of the Intron 34 *ROS1* breakpoint seen in our case. Most common *NPM1* breakpoints are reported to include Exons 1–4 [14], whereas the Intron 5 *NPM1* breakpoint identified in this case has not been described, to the best of our knowledge. Further, there is limited data to indicate sensitivity of *NPM1::ROS1* rearranged lung adenocarcinomas to crizotinib [15].

In conclusion, the nuclear staining pattern of ROS1 protein seen in this case is a unique pattern not previously reported that was consistent with *ROS1* gene rearrangement on FISH. Familiarity with different staining patterns is essential for screening cases and recommending appropriate follow‐up testing. In this case, the findings qualified this patient for *ROS1*‐directed tyrosine kinase inhibitor treatment. However, given the fact that this discovery took place in a reference laboratory setting (ARUP Laboratories, Salt Lake City, Utah), follow‐up information, including treatment, treatment response, and other metrics, such as initial response, progression‐free survival, and overall survival, were not available for this patient.

## Funding

No funding was received for this manuscript.

## Consent

No written consent has been obtained from the patient as there is no patient identifiable data included in this case report.

## Conflicts of Interest

The authors declare no conflicts of interest.

## Data Availability

The data that support the findings of this study are available from the corresponding author upon reasonable request.
